# Preexisting depression and COVID-19: a cohort study on the risk of susceptibility and hospitalization

**DOI:** 10.1186/s12888-023-05438-9

**Published:** 2023-12-13

**Authors:** Nastaran Nasirpour, Neda Esmailzadehha, Ahmad Hajebi, Ebtesam Savari, Behrooz Ghanbari, Abbas Motevalian

**Affiliations:** 1 Department of Epidemiology, School of Public Health, Iran University of Medical Sciences, Crossroads of Hemmat and Chamran Expressways, Tehran, Iran; 2 Research Center for Addiction and High-Risk Behaviors, Psychosocial Health Research Institute, Iran University of Medical Sciences, Tehran, Iran; 3 Department of Psychiatry, School of Public Health, Iran University of Medical Sciences, Tehran, Iran; 4 Trauma and Injury Research Center, Iran University of Medical Sciences, Tehran, Iran

**Keywords:** Major depressive disorder, COVID-19, Susceptibility, Severity, Hospitalization, Cohort studies

## Abstract

**Background:**

Depression can have negative effects on a person’s physical health. However, the available evidence on the risk of susceptibility to COVID-19 and its adverse outcomes in people with mental disorders, including depression, is limited and inconsistent. Therefore, we investigated the relationship between major depressive disorder (MDD) and the risk of susceptibility to COVID-19 infection and hospitalization. The data used in the study were obtained from the Employees’ Health Cohort Study of Iran (EHCSIR).

**Methods:**

We conducted a cohort study that included 3355 participants who had complete data on major depressive disorder at baseline assessment and two annual telephone follow-ups between January 2020 and March 2022. Trained psychologists used the Persian version of the Composite International Diagnostic Interview (CIDI-2.1) to identify major depressive disorders during the baseline assessment. We applied log binomial regression models to adjust for sociodemographic factors and background health conditions.

**Results:**

We found that 11.4% of participants had lifetime MDD and 7.3% had MDD in the past 12 months. During the pandemic, 26.1% of participants were infected with COVID-19, and 14.4% of those who were infected were hospitalized. The risk of susceptibility to COVID-19 infection was significantly higher among participants with lifetime MDD than among those without MDD (adjusted risk ratio (ARR) = 1.24, 95% CI: 1.06–1.47). However, lifetime MDD or 12-month MDD was not independently associated with hospitalization among COVID-19 cases.

**Conclusions:**

Preexisting major depressive disorder may increase the risk of susceptibility to COVID-19.

**Supplementary Information:**

The online version contains supplementary material available at 10.1186/s12888-023-05438-9.

## Background

Major depression, also known as major depressive disorder (MDD) or clinical depression, is a mood disorder that was ranked as the third cause of the burden of disease worldwide in 2008, and it has been projected that this disease will rank first by 2030 [1]. The Global Burden of Disease study from 1990 to 2017 found that the number of incident cases of MDD increased by 49.29% from 162 million to 241 million worldwide [2].

The lifetime prevalence of MDD is estimated to be approximately 5 to 17%, with an average of 12% [2]. The median 12-month prevalence of MDD based on 42 household surveys that utilize the Composite International Diagnostic Interview (CIDI) is 4.7% worldwide [3]. In Iran, the Iranian Mental Health Survey (IranMHS) utilizing the same instrument, the CIDI, found that the 12-month prevalence of MDD is 12.7% overall, with a higher prevalence in women (15.7%) than in men (10.7%) [4].

Individuals with depression are more likely to experience other health conditions or co-occurring disorders [5]. Depression can lead to an increased risk of infections due to increased levels of systemic inflammation and suppression or disruption of aspects of innate and adaptive immunity [6]. This suggests that MDD may affect the incidence and outcomes of coronavirus disease 2019 (COVID-19) through similar mechanisms.

COVID-19 is an ongoing global pandemic caused by severe acute respiratory syndrome coronavirus 2 (SARS-CoV-2). The COVID-19 pandemic has had a significant impact on the global population, particularly on disadvantaged groups. Among these groups, individuals affected by mental disorders, including depression, may have been particularly vulnerable [5]. From the beginning of the pandemic in 2019 to the end of 2022, COVID-19 caused 7.3 million reported deaths worldwide. The Institute for Health Metrics and Evaluation (IHME) COVID-19 models estimated as many as 17.9 million deaths. In Iran, the country experienced five waves of COVID-19 epidemic until the end of 2022, with the reported and estimated number of deaths being 144,000 and 260,000, respectively [7].

Since the first cases of the global outbreak of COVID-19, populations with specific risk factors have been largely described [8]. Identifying populations at risk for COVID-19 is important because there is no effective drug treatment for COVID-19. To provide specific prevention and treatment strategies, it is important to know the established risk factors [9]. Established risk factors for hospitalization include preexisting cardiovascular disease, obesity, diabetes, cancers, respiratory disease and weakened immune systems [9].

There have been few epidemiological studies on the relationship between preexisting mental disorders and COVID-19 susceptibility and severity [10–13]. Some studies have shown that patients with preexisting mental disorders have a higher risk of susceptibility to COVID-19 infection [10, 11]. A case‒control study conducted on 20% of the US population found that patients recently diagnosed with a mental disorder had a significantly higher risk of developing COVID-19. The study showed that the strongest effect was observed in patients with depression (adjusted odds ratio (AOR) = 7.64). However, others have not supported this association [12, 13]. However, most studies, including two nationwide studies in South Korea, have found that individuals with preexisting mental disorders are at a significantly higher risk of hospitalization for COVID-19 [12, 13].

Mental disorders may increase the risk of COVID-19 infection and its severity due to several factors, such as unhealthy lifestyle, low socioeconomic status, lack of awareness, and functional impairment [10, 12, 14–16]. MDD, which is one of the most common mental disorders worldwide [17], may also increase the risk of COVID-19 infection due to a low degree of chronic inflammation [18] or weaker self-care in people with depression [10].

On the other hand, in various studies, a significant number of patients with and survivors of COVID-19 have reported experiencing psychological issues, including depression [19, 20]. This suggests a possible two-way relationship between COVID-19 and mental health problems.

Most studies on the effect of mental disorders on COVID-19 are cross-sectional, which means that they are limited in their ability to draw causal conclusions. Therefore, we used baseline and follow-up data from the Employees’ Health Cohort Study of Iran (EHCSIR) to investigate the association of preexisting major depressive disorder (MDD) with susceptibility to COVID-19 infection and hospitalization.

## Methods

The EHCSIR is a prospective cohort study on the association of life style, social gradient, and work-related stressors with a wide range of non-communicable diseases. The recruitment and baseline assessments began in July 2017 and ended in March 2020 [21]. Annual telephone follow-up interviews have been conducted since January 2020. Questions regarding the COVID-19 outcomes were added to follow-up interviews from July 2020. In this study, we have used data regarding the existence of major depressive disorders from EHCSIR baseline assessments and data regarding the occurrence of COVID-19 from the two subsequent annual follow-ups.

### Participants

The EHCSIR study participants are 4886 public sector employees from schools, hospitals, and health centers affiliated with the Iran University of Medical Sciences and the Ministry of Health and Medical Education in Iran. The participation rate in annual telephone follow up interviews has been more than 99%. The EHCSIR participants were excluded if: (1) CIDI was not completed at baseline assessment, (2) less than 2 annual follow-up interviews for COVID-19.

### Ethical considerations

The research ethics committee of the Iran University of Medical Science approved the protocol of the current study with ethical code #IR.IUMS.REC.1401.716. This approval was separate from the EHCSIR protocol. All participants provided written informed consent before joining the study. The data collected from participants were kept confidential and only accessible to the main study investigators. The study was conducted in accordance with the Declaration of Helsinki [22], national guidelines, and regulations.

### Measurements

#### Baseline assessment of major depressive disorder

We employed the lifetime version of the Composite International Diagnostic Interview (CIDI, version 2.1) to assess the presence of the following psychiatric disorders: major depressive disorder, dysthymia, obsessive-compulsive disorder, and generalized anxiety disorder. The CIDI is a fully structured psychiatric diagnostic interview developed by the World Health Organization, designed for assessing psychiatric disorders according to DSM and ICD criteria. It is designed to be used by trained interviewers, who may include lay persons [4], with no clinical experience [23]. It has undergone extensive testing for reliability and validity in various settings and languages, and has demonstrated high reliability and validity for most of the diagnostic sections [4, 24, 25]. The Persian version of CIDI 2.1 has also been validated and has demonstrated satisfactory psychometric properties [26, 27]. Both the 12-month and lifetime Persian versions of CIDI have been extensively used in various studies, such as the Iranian Mental Health Survey (IranMHS) [27], the PERSIAN Youth Cohort [28], and the Employees’ Health Cohort Study of Iran. The CIDI 2.1 diagnostic interview is able to classify the depressive disorders based on type (single or recurrent) and severity (mild, moderate, and severe) [29].

During the baseline assessment of the EHCSIR, trained psychologists conducted interviews and the twelve-month MDD was recognized based on the most recent episode of the subjects with lifetime MDD. Single episode is declared if the participant had a major depressive episode in the past 12 months with no previous episode of MDD. In case that the participant had one or more episodes of MDD prior to the one in the past 12 months, it is considered a recurrent episode. In this study, the exposure is defined as any case of single or recurrent episode MDD with any level of severity in the past 12 months.

#### Sociodemographic factors and health conditions

Sociodemographic factors were extracted from the EHCSIR baseline dataset, including age, gender, educational attainment, workplace, job, wealth index, and marital status. Body mass index (BMI) was calculated using the weight and height of the participants at baseline, and obese subjects were defined as those with a BMI of 30 or higher. Current smoking was categorized as positive for participants who smoked one or more tobacco products on a daily or nondaily basis. Self-reported past medical history of the participants on the following disease conditions was also used: diabetes mellitus, coronary artery disease (CAD), hypertension, and anemia.

#### Follow-up procedures

Based on the EHCSIR protocol, annual telephone follow-up interviews have been conducted since January 2020, with the order of follow-ups determined by either the date of recruitment (for the first follow-up) or the date of the last successful follow-up interview (for subsequent follow-ups). The structured follow-up interviews included questions about any hospitalizations, new diseases, or diagnostic/therapeutic procedures over the past twelve months. If a participant reported any outcome, an outcome review process began, which included a second phone interview by a medical doctor and the collection of all relevant medical records. A medical history form was completed, including chief complaint, present illness, past medical history, drug history, and family history. The images of all relevant medical records were sent through WhatsApp application by participants or their close relatives. Two trained physicians, as members of the Outcome Review Committee, reviewed the medical documents to determine the final diagnosis.

#### Outcome assessment

Questions regarding the COVID-19 outcomes were added to the EHCSIR standard follow-up interviews from July 2020. The COVID-19 diagnosis was considered positive if the participant had one of the following conditions: (1) positive PCR test for SARS-CoV2, (2) radiologist report on lung CT scan that confirmed COVID-19, (3) a medical doctor diagnosed symptomatic COVID-19, and the person had close contact with a PCR positive case of COVDI-19, (4) a medical doctor diagnosed symptomatic COVID-19, and the patient received specific medication for COVID-19 treatment like dexamethasone and remdesivir.

Hospitalization due to COVID-19 was also considered. All relevant medical records were collected, and the diagnosis of COVID-19 was confirmed by the Outcome Review Committee. Follow-up data from July 2020 to March 2022 were used in the present study.

### Approach to missing data

Missing data were most commonly due to time constraints during the interview at cohort baseline or late baseline recruitment, resulting in less than two years of annual telephone follow-up. As these reasons can be considered missing completely at random (MCAR), a complete case analysis approach was applied to handle the missing data in the study.

### Statistical analysis

Descriptive statistics are presented as the mean ± standard deviation (SD) for continuous variables and as numbers (percentage) for categorical variables. Principal component analysis (PCA) was used to calculate the wealth index based on asset data.

The occurrence of COVID-19 in the past two years based on two annual follow-ups is considered as a binary outcome to assess the risk. Log binomial regression analysis was utilized to estimate the risk ratios of the association between MDD and susceptibility to COVID-19 infection. Additionally, log binomial regression analysis was used to identify the independent association of MDD with hospitalization among COVID-19 cases. Three models with no adjustment, minimal adjustment, and full adjustment were used in each of these two log binomial regression analyses. Adjusted risk ratios and their corresponding 95% confidence intervals (CIs) were presented. STATA version 14 was used for all analyses, and the significance level was set at 0.05.

While the main results are presented based on complete case analysis, we conducted sensitivity analysis under two scenarios. Firstly, we used multiple imputation method to fill the missing values of the exposure. Secondly, missing values of the outcome for participants who had not two annual follow-ups were filled by multiple imputation technique.

## Results

Out of the 4886 individuals who participated in the ECHSIR study, 3355 had complete data on both exposure and outcome and were included in this analysis. Table 1 presents the baseline characteristics of this study population. The average age of the participants when they joined the study was 42.8 years, with a standard deviation of 8.1 years, and the majority of them (62.6%) were female. Approximately 45.0% of the participants were health professionals, and 64.9% of them worked in hospitals or public health centers.

**Table 1:** The prevalence of the past twelve months and lifetime MDD by sociodemographic characteristics

		Total	Past twelve months MDD		Lifetime MDD	
			Cases	Prevalence (CI 95%)	Cases	Prevalence (CI 95%)
Gender	Male Female	1283 2072	65 179	5.1 (3.9–6.3) 8.6 (7.5–9.9)	104 278	8.1 (6.5–9.6) 13.4 (11.8–15.0)
Age (years)	$\le 34$ 35–44 45–54 $\ge 55$	556 1451 1046 302	40 115 67 22	7.2 (5.2–9.5) 7.9 (6.4–9.4) 6.4 (4.9–8.1) 7.3 (4.3–10.4)	62 174 112 34	11.2 (8.3–13.7) 12.0 (10.0-14.1) 10.7 (8.8–12.7) 11.3 (8.0-14.9)
Educational attainment	Primary or secondary High school diploma Associate Bachelor’s level Master’s level Doctoral level	347 693 309 1245 609 152	16 60 26 92 35 15	4.6 (2.5–6.4) 8.7 (6.6–10.8) 8.4 (5.3–13.0) 7.4 (6.1–8.8) 5.7 (3.7–7.8) 9.9 (6.1–14.8)	23 88 40 154 54 23	6.6 (4.2–9.3) 12.7 (10.4–15.0) 12.9 (9.3–18.1) 12.4 (10.4–14.4) 8.9 (5.9–11.7) 15.1 (10.1–21.9)
Job	Office work Academic Health Professional Service work	951 61 1540 700	76 7 110 48	8.0 (6.5–10.2) 11.5 (1.7–19.5) 7.1 (5.7–8.5) 6.9 (5.0-8.6)	109 10 183 71	11.5 (9.6–13.7) 16.4 (7.1–26.4) 11.9 (10.0-13.4) 10.1 (8.0-12.5)
Workplace	Educational\office Public health centers Hospitals	1177 434 1744	81 34 129	6.9 (5.4–8.7) 7.8 (6.0-10.5) 7.4 (6.0-8.7)	127 58 197	10.8 (9.1–12.9) 13.4 (10.1–17.2) 11.3 (9.7–13.0)
Marital status	Married Never married Ex-married	2725 427 203	174 41 29	6.4 (5.6–7.2) 9.6 (6.8–12.5) 14.3 (9.4–20.1)	284 62 36	10.4 (9.2–11.4) 14.5 (10.6–18.2) 17.7 (12.5–24.5)
Wealth index	Low Medium High	911 1664 779	67 126 51	7.4 (5.9–8.9) 7.6 (6.1–8.7) 6.5 (5.0-8.6)	108 197 77	11.9 (9.7–14.0) 11.8 (10.1–13.4) 9.9 (8.1–12.0)

The prevalence of MDDs among different sociodemographic groups, along with their 95% confidence intervals, can be found in Table 1. Out of 3355 individuals recruited, 382 (11.4%) had a lifetime diagnosis of MDD, and 244 (7.3%) had a diagnosis of MDD in the past twelve months.

During the two-year follow-up period, 876 cases of COVID-19 were identified. Of these patients, 64.6% were female, 7.5% were 55 years old and above, and 13.9% had lifetime MDD at baseline. In the lifetime MDD group, 122 people (31.9%) and in the non-MDD group, 754 people (25.4%) were identified as having COVID-19 infection (Table 2).

**Table 2:** Risk of susceptibility to COVID-19 infection and hospitalization among cohort participants

		Total	COVID-19 infection		COVID-19 Hospitalization	
			Cases	Risk (CI 95%)	Cases	Risk (CI 95%)
Gender	Male Female	1283 2072	310 566	24.2 (21.9–26.6) 27.3 (25.4–29.3)	53 73	17.1 (13.3–21.7) 12.9 (10.4–15.9)
Age (years)	$\le 34$ 35–44 45–54 $\ge 55$	556 1451 1046 302	156 392 262 66	28.1 (24.5–31.9) 27.0 (24.8–29.4) 25.0 (22.5–27.8) 21.9 (17.5–26.9)	15 53 45 13	9.6 (5.9–15.4) 13.5 (10.5–17.3) 17.2 (13.1–22.3) 19.7 (11.7–31.1)
Educational attainment	Primary or secondary High school diploma Associate Bachelor’s level Master’s level Doctoral level	347 693 309 1245 609 152	61 162 92 367 155 39	17.6 (13.9–22) 23.4 (20.4–26.7) 29.8 (24.9–35.1) 29.5 (27-32.1) 25.5 (22.1–29.1) 25.7 (19.3–33.2)	9 25 16 50 18 8	14.8 (7.8–26.1) 15.4 (10.6–21.9) 17.4 (10.9–26.6) 13.6 (10.5–17.5) 11.6 (7.4–17.7) 20.5 (10.5–36.2)
Job	Office work Academic Health Professional Service work	951 61 1540 700	195 19 482 162	20.5 (18.1–23.2) 31.1 (20.8–43.9) 31.3 (29-33.7) 23.1 (20.2–26.4)	24 4 71 24	12.3 (8.4–17.7) 21.1 (7.9–45.3) 14.7 (11.8–18.2) 14.8 (10.1–21.2)
Workplace	Educational\office Public health centers Hospitals	1177 434 1744	232 143 501	19.7 (17.5–22.1) 32.9 (28.7–37.5) 28.7 (26.6–30.9)	23 15 88	9.9 (6.7–14.5) 10.5 (6.4–16.7) 17.6 (14.5–21.2)
Marital status	Married Never married Ex-married	2725 427 203	690 120 66	25.3 (23.7–27) 28.1 (24-32.6) 32.5 (26.4–39.3)	98 12 16	14.2 (11.8–17) 10.1 (5.7–16.8) 24.2 (15.3–36.1)
Wealth index	Low Medium High	911 1664 779	230 445 200	25.2 (22.5–28.2) 26.7 (24.7–28.9) 25.7 (22.7–28.9)	28 64 34	12.2 (8.5–17.1) 14.4 (11.4–18) 17.0 (12.4–22.9)
Smoking	Yes No	424 2673	85 709	20.0 (16.5–24.1) 26.5 (24.9–28.2)	15 104	17.6 (10.9–27.3) 14.7 (12.2–17.5)
Obese	Yes No	805 2499	231 632	28.7 (25.7–31.9) 25.3 (23.6–27)	46 80	19.9 (15.2–25.6) 12.7 (10.3–15.5)
Anemia	Yes No	708 2363	182 603	25.7 (22.6–29.1) 25.5 (23.8–27.3)	31 86	17.0 (12.2–23.2) 14.3 (11.7–17.3)
Coronary Artery Disease	Yes No	79 2991	22 762	27.8 (19-38.8) 25.5 (23.9–27.1)	9 108	40.9 (22.5–62.3) 14.2 (11.9–16.8)
Diabetes mellitus	Yes No	153 2918	49 734	32.0 (25.1–39.9) 25.2 (23.6–26.8)	13 104	26.5 (16-40.7) 14.2 (11.8–16.9)
Hypertension	Yes No	275 2797	72 712	26.2 (21.3–31.7) 25.5 (23.9–27.1)	14 103	19.4 (11.8–30.3) 14.5 (12.1–17.3)
Past 12-month MDD	Yes No	244 3111	77 799	31.6 (26-37.7) 25.7 (24.2–27.2)	11 115	14.3 (8.1–24.1) 14.4 (12.1–17)
Lifetime MDD	Yes No	382 2973	122 754	31.9 (27.4–36.8) 25.4 (23.8–27)	21 105	17.2 (11.5–25) 13.9 (11.6–16.6)

After adjusting for gender, age, educational attainment, job, workplace, marital status, wealth index, smoking, obesity, anemia, CAD, diabetes mellitus, and hypertension, the risk of susceptibility to COVID-19 infection was significantly higher in those with lifetime MDD (adjusted risk ratio (ARR) = 1.4, 95% CI: 1.1–1.8) (Table 3). Similarly, in the group of patients with MDD in the past twelve months, 77 people (31.6%) and in the nonafflicted group, 799 people (25.7%) were identified as having COVID-19 (Table 2). The risk of susceptibility to COVID-19 infection in participants with MDD in the past twelve months was higher than that in nonsufferers (ARR = 1.3, 95% CI: 1.0-1.8) (Table 3). The details of log-binomial regression analyses are appeared in Supplementary Material (Tables S1 to S4).

The association between lifetime MDD (ARR = 1.3, 95% CI: 0.7–2.3) and MDD in the past twelve months (ARR = 1.0, 95% CI: 0.5–2.2) and hospitalization were not statistically significant (Table 3). The main results were robust in sensitivity analyses (See Table S5 in Supplementary Material).

**Table 3:** The association between baseline diagnosis of MDD and COVID-19 and hospitalization

Exposure		COVID-19 disease (n = 3355)			Hospitalized for COVID-19 (n = 876)		
		RR	95% CI	*P* value	RR	95% CI	*P* value
**Past twelve months MDD**							
	Unadjusted	1.24	1.02–1.50	< 0.05	1.00	0.55–1.80	0.980
	Model 1	1.22	1.01–1.48	< 0.05	1.15	0.66–2.03	0.622
	Model 2	1.19	0.97–1.46	0.10	1.02	0.48–2.15	0.960
**Lifetime MDD**							
	Unadjusted	1.28	1.10–1.49	< 0.005	1.40	0.93–2.11	0.104
	Model 1	1.27	1.08–1.48	< 0.005	1.37	0.82–2.30	0.232
	Model 2	1.24	1.06–1.47	< 0.01	1.26	0.71–2.24	0.707

Model 1: Adjusted for Age and Gender

Model 2: Adjusted for age, gender, educational attainment, job, workplace, marital status, wealth index, smoking, obesity, anemia, coronary artery disease, diabetes mellitus, and hypertension

## Discussion

Our findings from EHCSIR data indicate that individuals with a lifetime diagnosis of MDD or a diagnosis of MDD in the past 12 months have a significantly higher risk of susceptibility to COVID-19 infection than those without MDD. However, we found no evidence of an increased risk of hospitalization in participants with lifetime MDD or past twelve months of MDD among confirmed COVID-19 cases. In light of the adjusted risk ratio of susceptibility of COVID-19 in the present study (ARR = 1.24) and the 13% prevalence of 12-month MDD [4], the population attributable risk of depression for COVID-19 in Iran is approximately 3.8%. This means that 3.8% of COVID-19 cases in Iran can be attributed to MDD.

There is a debate on whether individuals with mood disorders are at a higher risk of contracting COVID-19. Two meta-analyses were conducted to investigate this association. The first meta-analysis included 18 studies on preexisting mental disorders, six of which had over 62 million participants. The results showed that patients with mood disorders had a higher risk of susceptibility to COVID-19 infection (odds ratio (OR) = 2.02; 95% CI: 1.08–3.76) [30]. On the other hand, the second meta-analysis that included 21 studies with over 91 million individuals found no association between mood disorders and COVID-19 susceptibility (OR = 1.27; 95% CI: 0.73–2.19; n = 65,514,469) [9]. It is important to note that only two of the 18 studies in the first meta-analysis and one study out of 21 studies in the second meta-analysis were from low-middle countries [9, 30]. The findings of studies may differ due to differences in demographic, socioeconomic, and clinical characteristics. Additionally, the biological and neural mechanisms of different mental disorders may differ, leading to a confounding association [30]. According to the analysis of the UK Biobank cohort, the risk of susceptibility to COVID-19 infection increases with the increase in the number of mental illnesses, but there is no significant relationship with the time that has passed since the diagnosis of a mental disorder [31]. A similar study in the US found that patients with recently diagnosed mental disorders (within the past year) are at high risk of susceptibility to COVID-19 infection (adjusted odds ratio (AOR) = 10.43), but the strength of this relationship is less for patients who have been diagnosed for more than a year (AOR = 1.48) [10].

The increased risk of susceptibility to COVID-19 infection in people with depressive disorders can be explained by several causal pathways. The first group of causal pathways includes MDD-related behaviors such as inhibition, apathy, lack of motivation, cognitive deficits, reduced awareness of risks, sleep dysregulation, habitual inactivity [9], neglect of self-protection [10], impairment in evaluating health information and obtaining preventive behaviors [32], stigma, and accessing health care [33]. The second group is comorbidities associated with MDD, which includes drug use disorders [34], diabetes, obesity, and cardiovascular diseases [9]. The third group is biological factors, which include inflammation [18], activation of the hypothalamic‒pituitary‒adrenal axis [35], changes in corticosteroids [36], drug therapy [9], and shared genetic susceptibility factors [37, 38]. However, social isolation, unemployment, and low interpersonal contact [9, 12] are possible mediators that may explain the lower risk of susceptibility to COVID-19 in MDD patients [12, 39].

In our study pre-existing MDD was not associated with increased risk of hospitalization among COVID-19 patients. Similarly, a phenome-wide association study found no association between mental disorders and hospitalization, ICU admission or mortality in 53,853 patients who were tested or diagnosed for COVID-19 at a large academic medical center [40]. However, there are several other studies that found a link between depressive disorders and COVID-19 hospitalization. A meta-analysis of eight studies with over 25 million participants found that preexisting mood disorders were associated with an increased risk of illness severity (OR = 1.34; 95% CI: 1.08–1.67) [30]. Another systematic review and meta-analysis reported that COVID-19 hospitalization is significantly higher in those with mood disorders (OR = 1.31; 95% CI; 1.12–1.53) [9]. The lack of motivation and difficulty in evaluating health information in patients with MDD can result in nonadherence to preventive behaviors, including tertiary prevention [10]. Additionally, the low economic status of this group can lead to delays in receiving medical care and going to the hospital [13]. Social determinants of health, such as economic insecurity, health literacy, and limited access to healthcare, have been identified as mediators of this association [9].

The lack of association between pre-existing MDD and hospitalization due to COVID-19 in our study may be explained by the characteristics of the study population. Since all of the subjects of the cohort study were public employees and the majority of them were working at hospitals or health centers, it is reasonable to assume that their health literacy, economic stability, and access to healthcare services are higher than those of the general population and are not affected by their mental disorders.

### Strengths and limitations

The study has several strengths. The most important strength is that the MDD was measured using a valid diagnostic tool, rather than relying on hospital records, self-report, or symptom scales. In contrast, similar published studies assessed preexisting MDD based on diagnosed, treated, or hospitalized cases. In the United States, the past 12-month and lifetime prevalence of MDD among adults were 10.4% and 20.6%, respectively. Only 12% of patients with MDD were hospitalized at some point in their life [41], which means that 10–15% of true MDD cases are among the MDD hospitalized group and 80–85% of them are among the nonexposed group. On average, 8% of the patients who are hospitalized due to other reasons are affected by MDD. In Iran, data from the national mental health survey (IranMHS) showed that 12.7% of the adult population had suffered from MDD in the past twelve months [4], and 41.2% of them had received any health services for their mental problem [42]. This means that if electronic health records are used, approximately 40% of adults with past twelve MDD will be found, and the remaining 60% will remain in the unexposed or healthy group. This nondifferential misclassification of exposure will cause underestimation of the investigated relationship.

Second, the previous studies, except for the UK Biobank Cohort Study, were retrospective cohort studies and had the usual limitations in measuring and adjusting confounding factors. However, in our study, all confounding variables, such as comorbidities, socioeconomic status, smoking, and obesity, were meticulously measured during the cohort baseline assessments.

Third, in this study, all subjects, both with and without MDD, were followed up annually twice. This follow-up was the most rigorous one for assessing the study hypothesis.

The study population, consisting mainly of healthcare professionals working at hospitals and public health centers, limits the generalizability of our findings. Therefore, caution should be taken when applying the results to the general population or all employees. This limitation may have contributed to the lack of association found between MDD and COVID-19 hospitalization, as discussed earlier.

## Conclusions

According to our study, individuals with preexisting major depressive disorder (MDD) are more susceptible to COVID-19 infection, but there is no significant association with hospitalization. Therefore, it is important to identify and address modifiable risk factors and prevent delays in providing healthcare to this population. Future research should investigate whether COVID-19 vaccination has different efficacies in individuals with MDD and whether COVID-19 infection can alter the natural course of MDD over time.

### Electronic supplementary material

Below is the link to the electronic supplementary material.

**Supplementary Material 1: Table S1**. Lifetime MDD and risk of COVID-19. **Table S2**. Past 12-month MDD and risk of COVID-19. **Table S3**. Lifetime MDD and risk of hospitalization among COVID-19 patients. **Table S4**. Past 12-month MDD and risk of hospitalization among COVID-19 patients. **Table S5** Sensitivity analyses of the association between baseline diagnosis of MDD and COVID-19 and hospitalization

## Data Availability

The datasets produced and analyzed during the current study are available upon request sent to the corresponding author.

## Abbreviations

AOR: Adjusted odds ratio
ARR: Adjusted risk ratio
BMI: Body mass index
CAD: Coronary artery disease
CIDI-2.1: Composite International Diagnostic Interview
CIs: Confidence intervals
COVID-19: Coronavirus disease 2019
EHCSIR: Employees’ Health Cohort Study of Iran
IHME: Institute for Health Metrics and Evaluation
MCAR: Missing completely at random
MDD: Major depressive disorder
PCA: Principal component analysis
SARS-CoV-2: Severe acute respiratory syndrome coronavirus
SD: Standard deviation

## References

[1] Keshavarz K, Hedayati A, Rezaei M, Goudarzi Z, Moghimi E, Rezaee M (2022). Economic burden of major depressive disorder: a case study in Southern Iran. BMC Psychiatry.

[2] Jain S, Gupta S, Li VW, Suthoff E, Arnaud A (2022). Humanistic and economic burden associated with depression in the United States: a cross-sectional survey analysis. BMC Psychiatry.

[3] Kohn R (2018). Epidemiology of common mental disorders. The Exercise Effect on Mental Health.

[4] Sharifi V, Amin-Esmaeili M, Hajebi A, Motevalian A, Radgoodarzi R, Hefazi M, Rahimi-Movaghar A (2015). Twelve-month prevalence and correlates of psychiatric disorders in Iran: the Iranian mental health survey. Arch Iran Med.

[5] Bertolini F, Witteveen AB, Young S, Cuijpers P, Ayuso-Mateos JL, Barbui C (2023). Risk of SARS-CoV-2 Infection, severe COVID-19 Illness and COVID-19 mortality in people with pre-existing mental disorders: an umbrella review. BMC Psychiatry.

[6] Ronaldson A, Arias de la Torre J, Sima R, Ashworth M, Armstrong D, Bakolis I, Hotopf M, Dregan A (2022). Prospective associations between depression and risk of hospitalisation for Infection: findings from the UK Biobank. Brain Behav Immu.

[7] COVID-19 projections. Institute for health metrics and evaluation, Seattle WA. 2023. https://covid19.healthdata.org/ Accessed 16 Oct 2023.

[8] Descamps A, Frenkiel J, Zarca K, Laidi C, Godin O, Launay O (2022). Association between mental disorders and COVID-19 outcomes among inpatients in France: a retrospective nationwide population-based study. J Psychiatr Res.

[9] Ceban F, Nogo D, Carvalho IP, Lee Y, Nasri F, Xiong J (2021). Association between Mood disorders and Risk of COVID-19 Infection, hospitalization, and death: a systematic review and Meta-analysis. JAMA Psychiatry.

[10] Wang QQ, Xu R, Volkow ND (2021). Increased risk of COVID-19 Infection and mortality in people with mental disorders: analysis from electronic health records in the United States. World Psychiatry.

[11] Taquet M, Luciano S, Geddes JR, Harrison PJ (2021). Bidirectional associations between COVID-19 and psychiatric disorder: retrospective cohort studies of 62 354 COVID-19 cases in the USA. Lancet Psychiatry.

[12] Jeon HL, Kwon JS, Park SH, Shin JY (2021). Association of mental disorders with SARS-CoV-2 Infection and severe health outcomes: nationwide cohort study. Br J Psychiatry.

[13] Lee SW, Yang JM, Moon SY, Yoo IK, Ha EK, Kim SY (2020). Association between mental Illness and COVID-19 susceptibility and clinical outcomes in South Korea: a nationwide cohort study. Lancet Psychiatry.

[14] Druss BG (2020). Addressing the COVID-19 pandemic in populations with Serious Mental Illness. JAMA Psychiatry.

[15] Yao H, Chen JH, Xu YF (2020). Patients with mental health disorders in the COVID-19 epidemic. Lancet Psychiatry.

[16] Goldberg X, Castaño-Vinyals G, Espinosa A, Carreras A, Liutsko L, Sicuri E (2022). Mental health and COVID-19 in a general population cohort in Spain (COVICAT study). Soc Psychiatry Psychiatr Epidemiol.

[17] Maj M (2011). When does depression become a mental disorder? British Journal of Psychiatry. Br J Psychiatry.

[18] Beurel E, Toups M, Nemeroff CB (2020). The bidirectional relationship of depression and inflammation: double trouble. Neuron.

[19] Badinlou F, Lundgren T, Jansson-Fröjmark M (2022). Mental health outcomes following COVID-19 Infection: impacts of post-COVID impairments and fatigue on depression, anxiety, and insomnia — a web survey in Sweden. BMC Psychiatry.

[20] Matsumoto K, Hamatani S, Shimizu E, Käll A, Andersson G (2022). Impact of post-COVID conditions on mental health: a cross-sectional study in Japan and Sweden. BMC Psychiatry.

[21] Rajabzadeh R, Janani L, Motevalian SA (2021). Effects of different invitation strategies on participation in a cohort study of Iranian public sector employees: a cluster randomized trial. BMC Med Res Methodol.

[22] World Medical Association (2013). World Medical Association Declaration of Helsinki: ethical principles for medical research involving human subjects. JAMA.

[23] Kessler RC, Ustün TB (2004). The World Mental Health (WMH) Survey Initiative Version of the World Health Organization (WHO) Composite International Diagnostic interview (CIDI). Int J Methods Psychiatr Res.

[24] Wittchen HU, Robins LN, Cottler LB, Sartorius N, Burke JD, Regier D. Cross-cultural feasibility, reliability and sources of variance of the Composite International Diagnostic interview (CIDI). The multicentre WHO/ADAMHA field trials. Br J Psychiatry. 1991;159:645–53, 658. 10.1192/bjp.159.5.645 1756340

[25] Wittchen HU (1994). Reliability and validity studies of the WHO–Composite International Diagnostic interview (CIDI): a critical review. J Psychiatr Res.

[26] Alaghband Rad J, Study of the reliability, validity, and feasibility of Farsi translation of the Composite International Diagnostic Interview (CIDI). In: Ahmadi Abhari SA, Malakooti K, Nasr Esfahani M, Razzaghi EM, Sadeghi M, Yasamy MT, editors. Mental health effects of Iraqi invasion of Kuwait in a war – torn population of Iran: an epidemiological and financial study of the consequences of the Kuwaiti oil well fire disaster in the aftermath of Iraqi invasion of Kuwait in 1991, United Nations Compensation Commission (UNCC) Monitoring and Assessment Project. Tehran - Iran: Islamic Republic of Iran Ministry of Health, Committee for assessment and follow up for damages resulting from the Iraq–Kuwait War; 2003.

[27] Rahimi-Movaghar A, Amin-Esmaeili M, Sharifi V, Hajebi A, Radgoodarzi R, Hefazi M, Motevalian A (2014). Iranian mental health survey: design and field procedures. Iran J Psychiatry.

[28] Khazaie H, Najafi F, Hamzeh B, Chehri A, Rahimi-Movaghar A, Amin-Esmaeili M, Moradi-Nazar M, Zakiei A, Komasi S, Pasdar Y (2018). Cluster analysis of psychiatric profile, its correlates, and using mental health services among the young people aged 15–34: findings from the first phase of Iranian youth cohort in Ravansar. Soc Psychiatry Psychiatr Epidemiol.

[29] Biesheuvel-Leliefeld KE, Kok GD, Bockting CL, de Graaf R, Ten Have M, van der Horst HE, van Schaik A, van Marwijk HW, Smit F (2016). Non-fatal Disease burden for subtypes of depressive disorder: population-based epidemiological study. BMC Psychiatry.

[30] Liu L, Ni SY, Yan W, Lu QD, Zhao YM, Xu YY (2021). Mental and neurological disorders and risk of COVID-19 susceptibility, Illness severity and mortality: a systematic review, meta-analysis and call for action. EClinicalMedicine.

[31] Yang H, Chen W, Hu Y, Chen Y, Zeng Y, Sun Y (2020). Pre-pandemic psychiatric disorders and risk of COVID-19: a UK Biobank cohort analysis. Lancet Healthy Longev.

[32] Shinn AK, Viron M (2020). Perspectives on the COVID-19 pandemic and individuals with serious mental Illness. J Clin Psychiatry.

[33] Heim E, Henderson C, Kohrt BA, Koschorke M, Milenova M, Thornicroft G (2019). Reducing mental health-related stigma among medical and nursing students in low- and middle-income countries: a systematic review. Epidemiol Psychiatr Sci.

[34] Rudenstine S, Espinosa A, Kumar A (2020). Depression and Anxiety Subgroups across Alcohol Use Disorder and Substance Use in a national epidemiologic study. J Dual Diagn.

[35] Donoso F, Cryan JF, Olavarría-Ramírez L, Nolan YM, Clarke G (2023). Inflammation, lifestyle factors, and the Microbiome-Gut-Brain Axis: relevance to Depression and Antidepressant Action. Clin Pharmacol Ther.

[36] Thibaut F (2019). Corticosteroid-induced psychiatric disorders: genetic studies are needed. Eur Arch Psychiatry Clin Neurosci.

[37] He Y, Yu R, Ren J (2021). The correlation between Psychiatric disorders and COVID-19: a narrative review. Psychiatr Danub.

[38] Nudel R, Wang Y, Appadurai V, Schork AJ, Buil A, Agerbo E (2019). A large-scale genomic investigation of susceptibility to Infection and its association with mental disorders in the Danish population. Transl Psychiatry.

[39] van der Meer D, Pinzón-Espinosa J, Lin BD, Tijdink JK, Vinkers CH, Guloksuz S (2020). Associations between psychiatric disorders, COVID-19 testing probability and COVID-19 testing results: findings from a population-based study. BJPsych Open.

[40] Salvatore M, Gu T, Mack JA, Sankar SP, Patil S, Valley TS (2021). A phenome-wide association study (PheWAS) of COVID-19 outcomes by race using the electronic health records data in Michigan medicine. J Clin Med.

[41] Citrome L, Jain R, Tung A, Landsman-Blumberg PB, Kramer K, Ali S (2019). Prevalence, treatment patterns, and stay characteristics associated with hospitalizations for major depressive disorder. J Affect Disord.

[42] Amin-Esmaeili M, Shadloo B, Rahimi-Movaghar A, Samimi Ardestani SM, Hajebi A, Khatibzadeh S (2022). Major depressive disorder in Iran: Epidemiology, Health Care Provision, utilization, and challenges. Arch Iran Med.

