# Supplementary material for: Preexisting depression and COVID-19: a cohort study on the risk of susceptibility and hospitalization
Source: BMC Psychiatry. 2023 Dec 13;23:942. doi: 10.1186/s12888-023-05438-9 (PMC10720084; doi:10.1186/s12888-023-05438-9)
Supplement: Supplementary file 1 — Supplementary Material 1: Table S1. Lifetime MDD and risk of COVID-19. Table S2. Past 12-month MDD and risk of COVID-19. Table S3. Lifetime MDD and risk of hospitalization among COVID-19 patients. Table S4. Past 12-month MDD and risk of hospitalization among COVID-19 patients. Table S5 Sensitivity analyses of the association between baseline diagnosis of MDD and COVID-19 and hospitalization [file 12888_2023_5438_MOESM1_ESM.docx]

# Supplementary Material

**Table S1**. Lifetime MDD and risk of COVID-19

|  | | **Unadjusted**  **RR (CI95%)** | **Model 1**  **RR (CI95%)** | **Model 2**  **RR (CI95%)** |
| --- | --- | --- | --- | --- |
| Lifetime MDD | Yes  No | 1.28^*^ (1.10-1.49)  1.0 | 1.27^*^ (1.08-1.48)  1.0 | 1.24^*^ (1.06-1.47)  1.0 |
| Gender | Female  Male | - | 1.11 (0.99-1.25)  1.0 | 0.99 (0.84-1.16)  1.0 |
| Age (years) | $\leq34$  35-44  45-54  $\geq55$ | - | 1.0  0.97 (0.83-1.13)  0.91 (0.77-1.08)  0.79 (0.62-1.02) | 1.0  1.02 (0.86-1.21)  0.99 (0.81-1.20)  0.90 (0.68-1.20) |
| Educational attainment | Primary or secondary  High school diploma  Associate  Bachelor’s level  Master’s level  Doctoral level | - | - | 1.0  1.57^*^ (1.17-2.13)  1.83^*^ (1.28-2.60)  1.85^*^ (1.32-2.60)  1.79^*^ (1.25-2.58)  1.63^*^ (1.0-2.64) |
| Job | Office work  Academic  Health Professional  Service work | - | - | 1.0  1.58 (0.95-2.65)  1.37^*^ (1.14-1.66)  1.29^*^ (1.01-1.64) |
| Workplace | Educational\office  Public health centers  Hospitals | - | - | 1.0  1.36^*^ (1.09-1.68)  1.24^*^ (1.03-1.49) |
| Marital status | Married  Never married  Ex-married | - | - | 1.0  1.05 (0.87-1.26)  1.32^*^ (1.06-1.64) |
| Wealth index | High Medium  Low | - | - | 0.90 (0.74-1.09)  0.92 (0.79-1.07)  1.0 |
| Smoking | Yes  No | - | - | 0.81 (0.64-1.01)  1.0 |
| Obese | Yes  No | - | - | 1.18^*^ (1.03-1.36)  1.0 |
| Anemia | Yes  No | - | - | 0.97 (0.84-1.13)  1.0 |
| CAD | Yes  No | - | - | 1.23 (0.84-1.79)  1.0 |
| Diabetes mellitus | Yes  No | - | - | 1.31^*^ (1.03-1.67)  1.0 |
| Hypertension | Yes  No | - | - | 1.05 (0.85-1.3)  1.0 |
| AIC |  | 1.1474 | 1.1473 | 1.1294 |
| BIC |  | -23374.7 | -23350.7 | -19846.8 |

**Table S2.** Past 12-month MDD and risk of COVID-19

|  | | **Unadjusted**  **RR (CI95%)** | **Model 1**  **RR (CI95%)** | **Model 2**  **RR (CI95%)** |
| --- | --- | --- | --- | --- |
| Past 12-month MDD | Yes  No | 1.24^*^ (1.02-1.50) 1.0 | 1.22^*^ (1.01-1.48)  1.0 | 1.19 (0.97-1.46)  1.0 |
| Gender | Female  Male | - | 1.12 (0.99-1.26)  1.0 | 1.04 (0.89-1.21)  1.0 |
| Age (years) | $\leq34$  35-44  45-54  $\geq55$ | - | 1.0  0.97 (0.83-1.13)  0.91 (0.77-1.08)  0.80 (0.62-1.02) | 1.0  1.03 (0.87-1.22)  1.01 (0.83-1.22)  0.90 (0.68-1.19) |
| Educational attainment | Primary or secondary  High school diploma  Associate  Bachelor’s level  Master’s level  Doctoral level | - | - | 1.0  1.52^*^ (1.13-2.04)  1.75^*^ (1.23-2.48)  1.79^*^ (1.28-2.50)  1.73^*^ (1.20-2.48)  1.65^*^ (1.03-2.63) |
| Job | Office work  Academic  Health Professional  Service work | - | - | 1.0  1.45 (0.86-2.42)  1.35^*^ (1.12-1.62)  1.26 (0.99-1.61) |
| Workplace | Educational\office  Public health centers  Hospitals | - | - | 1.0  1.38^*^ (1.11-1.71)  1.25^*^ (1.04-1.49) |
| Marital status | Married  Never married  Ex-married | - | - | 1.0  1.05 (0.88-1.27)  1.33^*^ (1.06-1.65) |
| Wealth index | High Medium  Low | - | - | 0.87 (0.72-1.05)  0.90 (0.78-1.06)  1.0 |
| Smoking | Yes  No | - | - | 0.82 (0.66-1.02)  1.0 |
| Obese | Yes  No | - | - | 1.18^*^ (1.3-1.36)  1.0 |
| Anemia | Yes  No | - | - | 0.97 (0.83-1.12)  1.0 |
| CAD | Yes  No | - | - | 1.23 (0.85-1.79)  1.0 |
| Diabetes mellitus | Yes  No | - | - | 1.35^*^ (1.05-1.73)  1.0 |
| Hypertension | Yes  No | - | - | 1.04 (0.84-1.29)  1.0 |
| AIC |  | 1.1484 | 1.1482 | 1.1310 |
| BIC |  | -23371.3 | -23347.5 | -19842.0 |

**Table S3**. Lifetime MDD and risk of hospitalization among COVID-19 patients

|  | | **Unadjusted**  **RR (CI95%)** | **Model 1**  **RR (CI95%)** | **Model 2**  **RR (CI95%)** |
| --- | --- | --- | --- | --- |
| Lifetime MDD | Yes  No | 1.40 (0.93-2.11)  1.0 | 1.37 (0.82-2.30)  1.0 | 1.26 (0.71-2.24)  1.0 |
| Gender | Female  Male | - | 0.76 (0.55-1.05)  1.0 | 0.67 (0.44-1.03)  1.0 |
| Age (years) | $\leq34$  35-44  45-54  $\geq55$ | - | 1.0  1.48 (0.86-2.55)  1.85^*^ (1.07-3.19)  2.23^*^ (1.12-4.42) | 1.0  1.16 (0.66-2.06)  1.31^*^ (0.71-2.42)  1.63^*^ (0.74-3.56) |
| Educational attainment | Primary or secondary  High school diploma  Associate  Bachelor’s level  Master’s level  Doctoral level | - | - | 1.0  1.47 (0.66-3.28)  1.60 (0.63-4.10)  1.16 (0.45-3.0)  1.20 (0.44-3.32)  1.62 (0.46-5.78) |
| Job | Office work  Academic  Health Professional  Service work | - | - | 1.0  1.17 (0.29-4.73)  0.76 (0.46-1.26)  0.69 (0.35-1.33) |
| Workplace | Educational\office  Public health centers  Hospitals | - | - | 1.0  1.78 (0.85-3.73)  2.87^*^ (1.59-5.18) |
| Marital status | Married  Never married  Ex-married | - | - | 1.0  0.91 (0.50-1.68)  2.25^*^ (1.24-4.07) |
| Wealth index | High Medium  Low | - | - | 1.87^*^ (1.09-3.21)  1.35 (0.86-2.12)  1.0 |
| Smoking | Yes  No | - | - | 1.04 (0.63-1.74)  1.0 |
| Obese | Yes  No | - | - | 1.48^*^ (1.02-2.14)  1.0 |
| Anemia | Yes  No | - | - | 1.34 (0.90-2.02)  1.0 |
| CAD | Yes  No | - | - | 2.17^*^ (1.15-4.09)  1.0 |
| Diabetes mellitus | Yes  No | - | - | 1.69^*^ (1.02-2.81)  1.0 |
| Hypertension | Yes  No | - | - | 1.04 (0.59-1.81)  1.0 |
| AIC |  | 0.8273 | 0.8287 | 0.8696 |
| BIC |  | -5201.0 | -5175.8 | -4217.5 |

**Table S4.** Past 12-month MDD and risk of hospitalization among COVID-19 patients

|  | | **Unadjusted**  **RR (CI95%)** | **Model 1**  **RR (CI95%)** | **Model 2**  **RR (CI95%)** |
| --- | --- | --- | --- | --- |
| Past 12-month MDD | Yes  No | 1.00 (0.55-1.80)  1.0 | 1.15 (0.66-2.03)  1.0 | 1.02 (0.48-2.15)  1.0 |
| Gender | Female  Male | - | 0.78 (0.56-1.07)  1.0 | 0.69 (0.45-1.05)  1.0 |
| Age (years) | $\leq34$  35-44  45-54  $\geq55$ | - | 1.0  1.47 (0.86-2.54)  1.84^*^ (1.06-3.18)  2.20^*^ (1.11-4.37) | 1.0  1.16 (0.66-2.05)  1.29 (0.70-2.37)  1.57 (0.72-3.45) |
| Educational attainment | Primary or secondary  High school diploma  Associate  Bachelor’s level  Master’s level  Doctoral level | - | - | 1.0  1.46 (0.66-3.27)  1.64 (0.64-4.21)  1.15 (0.44-3.0)  1.21 (0.44-3.34  1.62 (0.45-5.79) |
| Job | Office work  Academic  Health Professional  Service work | - | - | 1.0  1.18 (0.29-4.81)  0.77 (0.46-1.27)  0.69 (0.35-1.34) |
| Workplace | Educational\office  Public health centers  Hospitals | - | - | 1.0  1.77 (0.85-3.70)  2.86 (1.58-5.16) |
| Marital status | Married  Never married  Ex-married | - | - | 1.0  0.87 (0.47-1.61)  1.96 (1.10-3.50) |
| Wealth index | High Medium  Low | - | - | 1.82 (1.06-3.12)  1.33 (0.85-2.09)  1.0 |
| Smoking | Yes  No | - | - | 1.05 (0.63-1.76)  1.0 |
| Obese | Yes  No | - | - | 1.50 (1.03-2.18)  1.0 |
| Anemia | Yes  No | - | - | 1.36 (0.90-2.04)  1.0 |
| CAD | Yes  No | - | - | 2.27 (1.20-4.29)  1.0 |
| Diabetes mellitus | Yes  No | - | - | 1.70 (1.02-2.84  1.0 |
| Hypertension | Yes  No | - | - | 1.02 (0.58-1.77)  1.0 |
| AIC |  | 0.8283 | 0.8301 | 0.8700 |
| BIC |  | -5200.0 | -5174.6 | -4217.2 |

**Table S5** Sensitivity analyses of the association between baseline diagnosis of MDD and COVID-19 and hospitalization

| Multiple imputation of exposure* | | | | |  | | |
| --- | --- | --- | --- | --- | --- | --- | --- |
| **Exposure** | | **COVID-19 disease**  **(n=3499)** | | | **Hospitalized for COVID-19**  **(n=900)** | | |
|  |  | **RR** | **95% CI** | **P value** | **RR** | **95% CI** | **P value** |
| **Past twelve months MDD** | |  | | | | | |
|  | Unadjusted | 1.24 | 1.02-1.50 | < 0.05 | 1.01 | 0.57-1.78 | 0.98 |
|  | Model 1 | 1.22 | 1.00-1.47 | < 0.05 | 1.04 | 0.59-1.84 | 0.88 |
|  | Model 2 | 1.22 | 0.99-1.49 | 0.06 | 1.05 | 0.56-1.92 | 0.87 |
| **Lifetime MDD** | |  | | | | | |
|  | Unadjusted | 1.25 | 1.07-1.47 | < 0.01 | 1.23 | 0.80-1.88 | 0.34 |
|  | Model 1 | 1.24 | 1.05-1.46 | < 0.01 | 1.28 | 0.83-1.96 | 0.27 |
|  | Model 2 | 1.26 | 1.06-1.49 | < 0.01 | 1.20 | 0.77-1.89 | 0.42 |

## Multiple imputation of outcome**

| **Exposure** | | **COVID-19 disease**  **(n=4072)** | | |
| --- | --- | --- | --- | --- |
|  |  | **RR** | **95% CI** | **P value** |
| **Past twelve months MDD** | |  | | |
|  | Unadjusted | 1.17 | 0.95-1.43 | 0.14 |
|  | Model 1 | 1.22 | 1.00-1.47 | 0.06 |
|  | Model 2 | 1.16 | 0.94-1.43 | 0.17 |
| **Lifetime MDD** | |  | | |
|  | Unadjusted | 1.19 | 1.01-1.39 | < 0.05 |
|  | Model 1 | 1.17 | 1.00-1.38 | < 0.05 |
|  | Model 2 | 1.20 | 1.01-1.41 | < 0.05 |

Model 1: Adjusted for Age and Gender

Model 2: Adjusted for age, gender, educational attainment, job, workplace, marital status, wealth index, smoking, obesity, anemia, coronary artery disease, diabetes mellitus, and hypertension

*For individuals who had not completed CIDI interview in baseline assessments, past 12-month and lifetime MDD were imputed using STATA mi command

**For study participants who had not the chance to complete two annual follow-ups for COVID-19 outcome assessment, COVID-19 data were imputed using multiple imputation method
